# *E le Saua le Alofa* (Love shouldn’t hurt): exploring the acceptability, feasibility and potential impact of a co-developed intervention to prevent violence against women in Samoa

**DOI:** 10.1186/s12889-026-27013-z

**Published:** 2026-03-24

**Authors:** Jenevieve Mannell, Papali’i Ene Isaako Hosea, Fa’afetai Alisi-Fesili, Siliniu Lina Chang, Helen Tanielu, Hattie Lowe, Faith Miller, Andrew Copas

**Affiliations:** 1 UCL Institute for Global Health, London, UK; 2 Samoa Victim Support Group, Apia, Samoa; 3 Independent Consultant, Apia, Samoa; 4 National University of Samoa, Apia, Samoa

**Keywords:** Acceptability and feasibility study, Intimate partner violence, Prevention, Co-design, Samoa

## Abstract

Women in Samoa experience a high rate of intimate partner violence (IPV) shaped in part by a history of colonisation and its influence on gender roles and lived experiences. As part of the EVE Project to improve the evidence base for preventing IPV in high-prevalence settings, we co-developed an intervention with 30 community-based researchers (CBRs) from 10 villages, drawing from evidence-based modules previously developed and evaluated in other neo-colonial contexts. We assessed the pilot intervention’s acceptability, feasibility and potential impact when delivered to groups of 15 men and 15 women in 10 participating villages, using a mixed methods before (baseline) and after (endline) study design. We collected a baseline (*N* = 289) and endline survey (*N* = 229); ‘stories of change’ interviews with participants (*N* = 151); in-depth interviews (*N* = 14) with CBRs, and intervention evaluation workbooks from CBRs (*N* = 18). The acceptability of the intervention was assessed through retention rates and participant interviews, while feasibility was assessed by interviews with CBRs, and observations of survey implementation. The potential impact of the intervention on IPV experience and perpetration, and on gender views was explored via the analysis of survey data. Results suggest a reduction in IPV experience and perpetration at endline compared to baseline, but associations were non-significant. Women’s belief of non-equitable norms was also lower at endline (β = − 0.54, 95% CI − 0.88 to − 0.21, *p* = 0.005). Attendance was high overall (although men’s acceptability was challenging in some villages). Participants shared positive feedback on the delivery by CBRs and careful adaptation to *fa’a Samoa* (‘the Samoan way’). Further research aligned with the Indigenous context of Samoa is required to assess its effectiveness. The study was registered retrospective to the pilot but prior to analysis as ISRCTN17319820 on 14 March 2024.

**Supplementary Information:**

The online version contains supplementary material available at 10.1186/s12889-026-27013-z.

## Background

Women in Samoa experience high rates of intimate partner violence (IPV), with 39.6% of women reporting an experience of IPV in their lifetime and 31.4% in the past 12 months, according to official statistics collected by the Samoan government in 2019-2020 [1]. This is a structural problem driven in part by a legacy of colonialism in the Pacific region, which reshaped gender norms according to the ideals of 18th century missionaries [2]. While Christianity was widely embraced by Samoans [3], there has been a recent backlash against the imposition of frameworks arising from high-income countries, which are often misaligned with local cultural practices and Pacific epistemologies [4]. Addressing the high rates of IPV therefore requires structural solutions that resonate with the local sociocultural and Christian context of present-day Samoa for both pragmatic and ethical reasons. Towards this goal, this paper explores the results of a structural IPV prevention intervention co-produced with Samoan communities as an approach grounded in local epistemologies.

Across low- and middle-income countries (LMICs), structural interventions that seek to address IPV by confronting legacies of power have shown some promise. For example, *SASA!*, a community mobilisation programme in Kampala, Uganda, addressed gendered power imbalances by engaging community activists, religious and traditional in reflective dialogue about gender norms, achieving reductions in both the perpetration and experience of IPV [5]. Other interventions have successfully addressed economic power at the level of households or relationships, which shapes who controls resources, who makes decisions about spending, and whose needs and priorities are met. For example, *Stepping Stones and Creating Futures* and its more recent adaptation for mental health [6], combined gender-transformative group work with livelihood strengthening among youth living in urban informal settlements in South Africa, and was effective in reducing men’s self-reported perpetration of physical and sexual intimate partner violence, while also improving women’s economic status [7]. In Côte d’Ivoire, interventions combining group savings for women with gender dialogue groups for couples showed reduced economic IPV compared to savings alone [8].

Despite the growing number of structural interventions, there remains a significant gap in rigorously evaluated IPV prevention strategies in Samoa, and the Pacific more broadly. Few Pacific programmes have had robust impact evaluations, particularly ones that explicitly tackle the intertwined effects of colonial histories, religion, customary authority, and gender inequality. As a result, evidence on what works (and for whom) in Samoan and Pacific contexts remain thin, particularly on prevention [9].

To help fill this gap, researchers at University College London (UCL) partnered with the Samoa Victim Support Group (SVSG) and the National University of Samoa (NUS) to co-design a violence prevention intervention as part of the EVE Project (“Evidence for Violence prevention in the Extreme”)—a broader programme of research to develop the evidence on violence prevention in high prevalence settings [10]. Our intervention, referred to locally as *E le Saua le Alofa* (Love Shouldn’t Hurt), explicitly centred Samoan customs and perspectives, local understandings of violence, and community priorities in its development. In this paper, we share the results of a pilot of the intervention completed in 2023, and discuss the implications for prevention of IPV in the Pacific.

### Setting

Samoa is an independent Pacific Island nation situated near Fiji and Tonga, with a population estimated at 198,414, the majority of whom reside in small rural settlements [1]. Roughly a quarter of Samoans live in the capital, Apia, which itself is organised into village-based communities. With a cultural history spanning more than 3,500 years, Samoa retains many longstanding traditions within contemporary life. These include the *Matai* chiefly system at the village level, a collectivist ethos that emphasises community over the individual, and extended kinship networks known as *aiga potopoto* [11].

During the early 20th century, Samoa was first colonised by Germany (1900–1914) before coming under New Zealand administration (1914–1962). Close ties with New Zealand remain, especially through migration and the regular flow of financial remittances from the Samoan diaspora [12]. Samoa is made up of 11 administrative districts across four inhabited islands, the largest being Upolu and Savai’i. It has 265 rural villages, in addition to 71 within Apia. Villages generally range from 350 to 800 inhabitants. Some are deeply rooted in history and tradition, while others are newer communities without the same features of customary organisation (i.e., Matai leadership, village councils, or communal land). Villages on Savai’i tend to be more traditional than those on Upolu, and there are also notable differences between rural and urban areas in terms of population size, employment, education, and household income.

## Previous work to co-design an intervention

From 2020 until 2023, the EVE Project team (composed of staff from SVSG, NUS, and UCL) co-designed the *E le Saua le Alofa* intervention collaboratively with 30 CBRs from 10 Samoan villages. SVSG, as the implementing partner for the project, selected ten villages stratified according to the number of cases of violence reported to the organisation since 2005, village structure (traditional/ urbanised), and location (urban/rural). SVSG then selected three individuals from each village from their existing networks: one man, one woman and one elder. The co-design process with CBRs was guided by an international advisory committee of global experts in IPV prevention interventions and evaluation, and a Samoan advisory group composed of a diverse group of stakeholders intimately familiar with the Samoan context.

As part of the co-design process, CBRs participated in tailored workshops facilitated by SVSG, NUS and UCL to develop research skills relevant to the project and strengthen their capacity to engage in the research. With guidance and support from SVSG and UCL, CBRs conducted peer interviews with members of their village about community responses to VAW and organised a village survey to assess risk and protective factors for VAW. We co-analysed interview and survey data during CBR workshops and developed a comprehensive Theory of Change (ToC) about how VAW could be prevented in Samoa together with CBRs.

We then used the ToC design to develop the various components of the intervention. We identified four drivers of violence that could feasibly be tackled through an intervention, namely: abuse of power by men, gendered social norms, poor communication between couples, and family conflict arising from alcohol abuse, financial stress and previous experiences of child abuse [13]. Through a facilitated discussion with CBRs, the team decided on six intervention components to address these drivers, namely: (1) addressing gendered power relations, (2) developing skills for healthy relationships (recognising triggers, positive time) (3) developing effective communication skills, (4) positive parenting, (5) supporting livelihoods, and (6) providing support for IPV survivors. A full description of each component is provided as supplementary material.

**Fig. 1:**
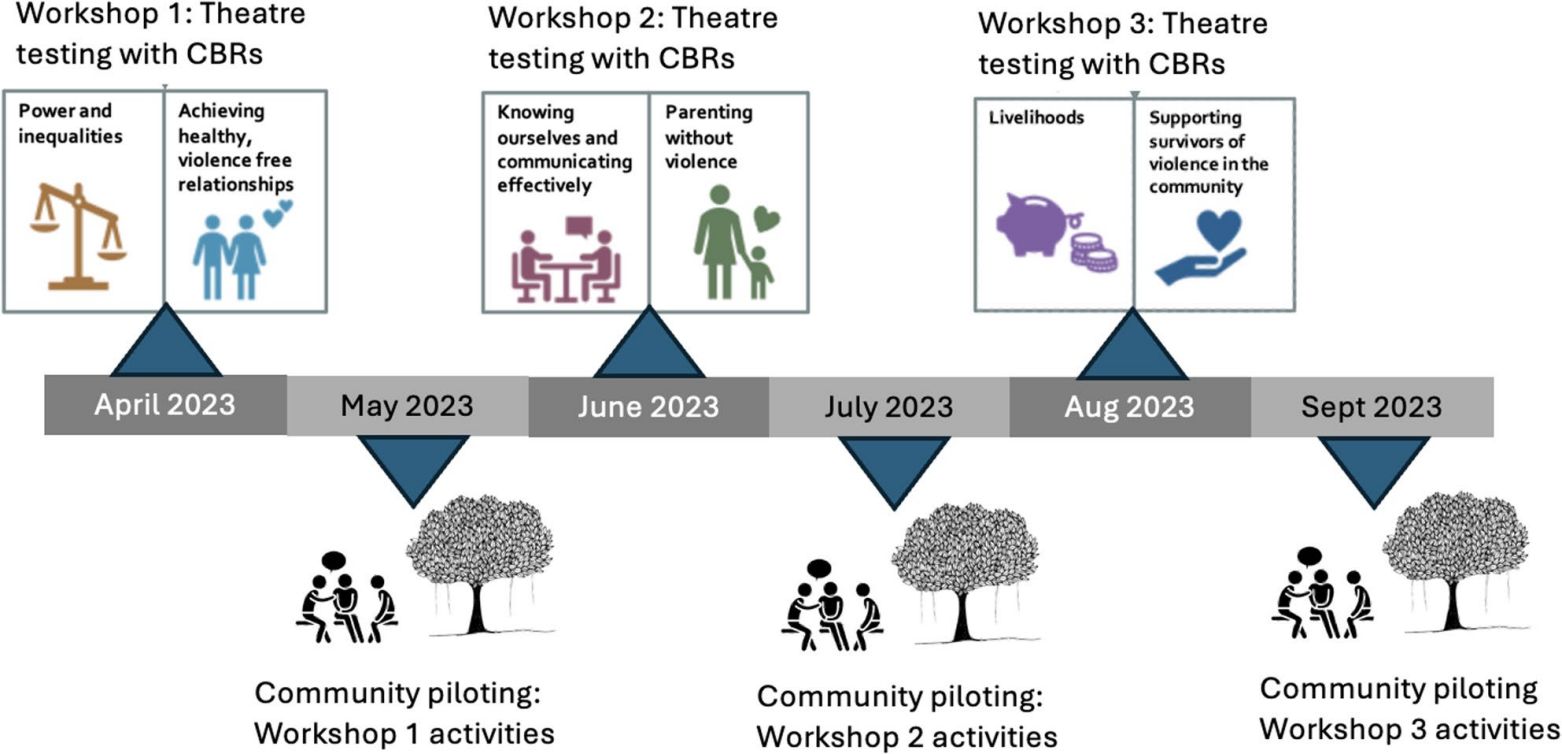
Iterative intervention theatre testing and piloting

SVSG and UCL subsequently completed a systematic evidence review to identify all intervention evaluations (randomised controlled trials or quasi-experimental studies) of interventions that aimed to prevent VAWG, published between 2000 and 2018. We reviewed 104 interventions, including only those classified as either “promising” or “effective.” We extracted intervention components and organised each thematically to create a “menu” of intervention activities. We then selected one activity from each relevant theme based on our knowledge of the Samoan context to include in a preliminary intervention manual (Fig. 1). We drew on select activities from well-regarded interventions in the Democratic Republic of Congo, Rwanda, South Africa, Tajikistan, Uganda, and Tanzania including: Stepping Stones [14], Stepping Stones Creating Futures [7], Engaging Men in Accountable Practices [15], MAISHA [16], Indashyikirwa [17], Bandebereho [18], Parenting for Respectability [19], and Zindagii Shoista [20].

## Piloting the intervention: *E le Saua le Alofa*

We used the ADAPT-ITT methodology of theatre testing [21] to live-test activities with CBRs and integrate their feedback into the final intervention manual. We delivered the six intervention components in three 2-day workshops. On average, the length of intervention components was 6-hours long, representing 36 hours of contact time with participants, delivered in 6 days.

After each 2-day theatre testing workshop with CBRs, facilitators adapted and implemented activities in the 10 participating villages, supported by the CBRs. This draws on a participatory action research approach, which involves iterative cycles of reflection and action by villages instead of delivering the full 6-day intervention in a single block [22]. Lessons learned during each 2-day workshop were carried through into subsequent sessions. The implementation of all intervention activities took place between March and September 2023.

## Methods of data collection

**Fig. 2:**
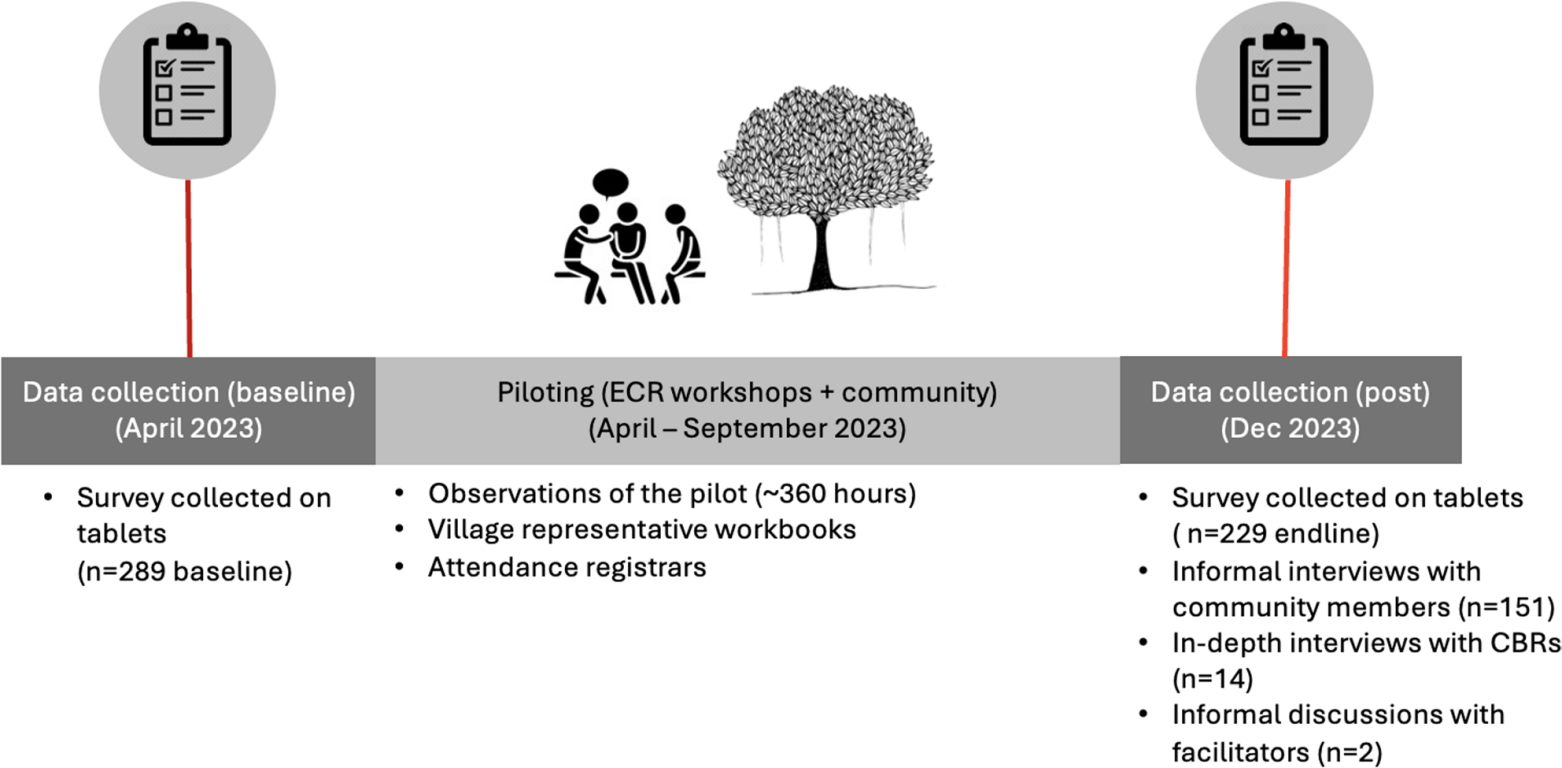
Data collected as part of the pilot evaluation

We conducted a mixed methods pilot acceptability and feasibility study, with an endline survey 44–48 weeks after the baseline survey. CBRs from each of the 10 villages selected 30 members of their village to participate in the intervention. In selecting participants, CBRs were asked to ensure an equal number of women and men distributed across age and village status (within the Matai hierarchal system of titled chiefs), but this was neither authoritative nor prescriptive and CBRs were expected to make independent decisions about who they thought should participate based on their research training and knowledge of their village context. Any community member was eligible to participate if they resided in one of the participating villages, were 18 years of age or older, and were willing to attend all six sessions. The study offered a small sum to participate ($20 tala per 1-day session; ~6GBP) to encourage participation and compensate participants for not participating in other livelihood activities (i.e., selling vegetables). Community members signed a consent form and completed the baseline survey on day one. The survey was self-administered on tablets with team members standing nearby to assist participants with any technical difficulties or to answer questions. Qualitative interviews following a ‘stories of change’ methodology [23] were completed in Samoan with 151 community participants (64 men; 87 women) at the same time as the endline survey (8–12 weeks after the end of the intervention). A separate round of qualitative interviews was completed with 14 CBRs about their experience of implementing the intervention and logistical successes/challenges. CBRs also completed a workbook during the intervention, which was submitted to the project team (Fig. 2).

### Outcomes

Acceptability was assessed quantitatively by retention rates monitored through attendance registers taken at the beginning of each session [24]. Acceptability was also assessed qualitatively during in-depth interviews with participants who were asked what they liked and did not like about the intervention and how it was implemented, and if they had any examples of changes that had come about because of the community’s participation. Members of the facilitation team carried out these interviews in Samoan during a dedicated evaluation session after the end of the intervention, and the data were transcribed into English in Excel for analysis. All sensitive or identifying information was removed from the data file.

Feasibility was assessed through interviews with CBRs conducted by an independent researcher familiar with the intervention but not directly involved in its delivery. CBRs were also asked to complete project workbooks, which asked questions about successes/challenges in implementing the intervention in their village. Workbooks were collected at the end of the intervention and translated and transcribed into English. Feasibility was also assessed through direct observation of the intervention by the evaluation team who attended at least one intervention day in each of the ten villages and took detailed notes, and multiple in-depth discussion between the evaluation team and the intervention facilitators.

While not the main aim of the evaluation, we also assessed the intervention’s impact on IPV outcomes to ensure that there was no clear evidence of harm to the population (i.e., by increasing violence) that would suggest we should not progress to a larger evaluation. Outcomes included women’s experience of physical, sexual, emotional and economic violence, men’s perpetration of physical, sexual, emotional and economic violence in the past six months, and views on gender norms (Table 1). Physical, sexual and emotional violence were assessed by the same scale used by the 2019-20 Samoan Demographic and Health Multiple Indicator Cluster Survey (DHS-MICS) to ensure comparability with national statistics, including six items on physical violence (e.g. punching, kicking, choking), three items on sexual violence (e.g. forced sex), and four items on emotional violence (e.g. humiliation, insults). Economic violence included five items from the Adapted Scale of Economic Abuse (ASEA) [25] adapted for the local context. Any positive response to any type of violence was considered evidence of violence experience/ perpetration and coded as binary (either having experienced/perpetrated VAW or not having experienced/perpetrated VAW).

Views on gender norms were assessed using the Gender Equitable Men (GEM) scale, which captures agreement with statements reflecting gender-inequitable views and is summarised as an additive composite score [26]. We drew on extensive qualitative work completed as part of the EVE Project to adapt the GEM scale for the Samoan context, maintaining eight items from the original tool and adding one new item (‘I believe it is god’s will that a man is the head of the family.’).

Questionnaires were self-completed at baseline and endline on a tablet using Kobo Toolbox. A field worker associated with SVSG was present to support participants or refer to counselling support if necessary.

**Table 1:** Outcomes collected at baseline and endline to assess potential impact of the intervention

Outcome	Source	Indicator	Number of items	Cronbach alpha	Methods of scaling	Hypothesized direction of change
Physical IPV past 6 months (experienced by women or perpetrated by men)	DHS-MICS	One or more episode of physical violence in the past 6 months	6	Not applicable	Binary	Decrease
Sexual IPV past 6 months (experienced by women or perpetrated by men)	DHS-MICS	One or more episode of sexual violence in the past 6 months	3	Not applicable	Binary	Decrease
Emotional IPV past 6 months (experienced by women or perpetrated by men)	DHS-MICS	One or more episode of emotional violence in the past 6 months	4	Not applicable	Binary	Decrease
Economic IPV past 6 months (experienced by women or perpetrated by men)	ASEA	One or more episode of economic violence in the past 6 months	5	Not applicable	Binary	Decrease
Views on acceptance of violence	GEM	Agreement with at least one question accepting violence	5	Women = 0.7 Men = 0.7	Score (range 0–16)	Decrease
Views of gender roles	GEM	Agreement with at least one question accepting gender roles	4	Women = 0.9 Men = 0.8	Score (range 0–12)	Decrease

*Abbreviations*: *ASEA* Adapted Scale of Economic Abuse scale, *DHS-MICS* Demographic and Health Survey and Multiple Indicator Cluster Survey, *GEM* Gender Equitable Men scale

### Statistical analyses

Quantitative survey data were analysed in STATA 18. While many participants completed surveys at baseline and endline, we were unable to reliably match surveys to analyse change within participant and not all participants completed the endline survey. We present socio-demographic data of participants at baseline and endline.

To quantify differences in outcomes between timepoints (baseline or endline), we ran logistic regressions (*logistic* STATA command for IPV experience and perpetration, and linear regressions (regress STATA command) for views on acceptance of violence and gender norms. We adjusted models for variables identified in the literature as potential confounders (age and education). To account for clustering at the village level, we used cluster-robust standard errors (vce(cluster)). The difference between baseline and endline surveys was reported as odds ratios with 95% confidence intervals and presented at co-author meetings for interpretation within the Samoan context.

### Ethics

Ethical approval for the study was granted by UCL Research Ethics Committee (9663.002) and the National University of Samoa (18.08.2022). Participants provided written informed consent prior to the start of the intervention, and again at the start of the survey. Additional consent was also sought for the qualitative interviews.

## Results

CBRs invited 30 individuals (15 men and 15 women) to participate in the intervention. Of the 300 individuals invited, 289 completed a baseline survey. The socio-demographics from the survey show a balance between age groups, particularly among men at baseline. This is notable because of the general lack of inclusion of younger people (18–24) in community activities in Samoa. Those aged 25–39 was the largest group of participants, largely reflecting the number of women participating who belonged to this age group (34.8%) and the demographics of Samoan communities more broadly. The majority had completed secondary education (71.3%), were living with partners (69.9%) and held a Matai title (42.2%) (Table 2).

**Table 2:** Socio-demographic data of participants

	Baseline			Endline		
	*N* (%)	Women (%)	Men (%)	*N* (%)	Women (%)	Men (%)
	289 (100%)	161 (55.7%)	128 (44.3%)	229 (100%)	140 (61.1%)	89 (38.9%)
Age *(missing)*	*2 (0.7%)*	*2 (1.2%)*	*0 (0%)*	*4 (1.8%)*	*2 (1.4%)*	*2 (2.3%)*
18–24 (youth)	37 (12.8%)	8 (5.0%)	29 (22.7%)	31 (13.5%)	11 (7.9%)	20 (22.5%)
25–39	83 (27.2%)	56 (34.8%)	27 (21.1%)	47 (20.5%)	34 (24.3%)	13 (14.6%)
40–49	70 (22.7%)	44 (27.3%)	26 (20.3%)	68 (29.7%)	43 (30.7%)	25 (28.0%)
50–59	59 (19.7%)	32 (19.9%)	27 (21.1%)	55 (24.0%)	38 (27.1%)	17 (19.1%)
60+	38 (12.9%)	19 (11.8%)	19 (14.8%)	24 (10.5%)	12 (8.6%)	12 (13.5%)
Education *(missing)*	*4 (6.2%)*	*2 (1.2%)*	*2 (1.6%)*	*8 (3.5%)*	*4 (2.9%)*	*4 (4.5%)*
Never attended	1 (0.4%)	0 (0.0%)	1 (0.8%)	1 (0.4%)	1 (0.7%)	0 (0%)
Early childhood education	3 (1.3%)	2 (1.2%)	1 (0.8%)	2 (0.9%)	0 (0.0%)	2 (2.2%)
Primary	19 (6.5%)	4 (2.5%)	15 (11.7%)	16 (7.0%)	7 (5.0%)	9 (10.1%)
Secondary	218 (71.3%)	131 (81.4%)	87 (67.9%)	166 (72.5%)	104 (74.3%)	62 (69.7%)
Higher	44 (14.3%)	22 (13.7%)	22 (17.2%)	36 (15.8%)	24 (17.1%)	12 (13.5%)
Relationship *(missing)*	*12 (4.2%)*	*5 (3.1%)*	*7 (5.5%)*	*5 (2.2%)*	*2 (1.4%)*	*3 (3.4%)*
Partnered, living together	202 (69.9%)	125 (77.7%)	77 (60.2%)	151 (65.9%)	95 (67.9%)	56 (62.9%)
Partnered, not living together	9 (3.1%)	6 (3.7%)	3 (2.3%)	7 (3.1%)	7 (5.0%)	0 (0.0%)
No relationship	66 (22.8%)	25 (15.5%)	41 (32.0%)	66 (28.8%)	36 (25.7%)	30 (33.7%)
Status *(missing)*	*53 (18.3%)*	*31 (19.3%)*	*22 (17.2%)*	*23 (10.0%)*	*19 (13.6%)*	*4 (4.5%)*
* Matai* (Village chief)	122 (42.2%)	76 (47.2%)	46 (35.9%)	113 (49.3%)	73 (52.1%)	40 (44.9%)
Nofotane/ Faiava*	48 (16.6%)	36 (22.4%)	12 (9.4%)	37 (16.2%)	28 (20.0%)	9 (10.1%)
Religious leader (e.g. minister, deacon)	4 (1.4%)	2 (1.2%)	2 (1.6%)	3 (1.3%)	0 (0.0%)	3 (3.4%)
Partner of religious leader	1 (0.4%)	1 (0.6%)	0 (0.0%)	4 (1.8%)	4 (2.9%)	0 (0.0%)
Untitled	61 (21.1%)	15 (9.3%)	46 (35.9%)	49 (21.4%)	16 (11.4%)	33 (37.1%)

Missing data is provided for each category and has been *italicised*

*Nofotane (woman)*/ Faiava* (man) is a stigmatised social identity signifying a woman/man who currently reside through marriage in a different village than where they were born

At endline, fewer respondents responded to the survey (*n* = 229/289), however the demographic profile of participants remains largely unchanged. The proportion of participants with secondary education (72.5%), living with partners (65.9%) and Matai status (49.3%) remained similar to baseline figures. More women than men completed the survey at both baseline (55.4%) and endline (61.1%).

### Acceptability

The attendance rates of community participants provide some indication of the acceptability of the intervention. Higher numbers of women participated in the intervention than men. Reasons reported to the project team included men travelling to New Zealand for work or healthcare, or men taking up irregular work opportunities on plantations on the same day the intervention was scheduled to take place.

**Table 3:**
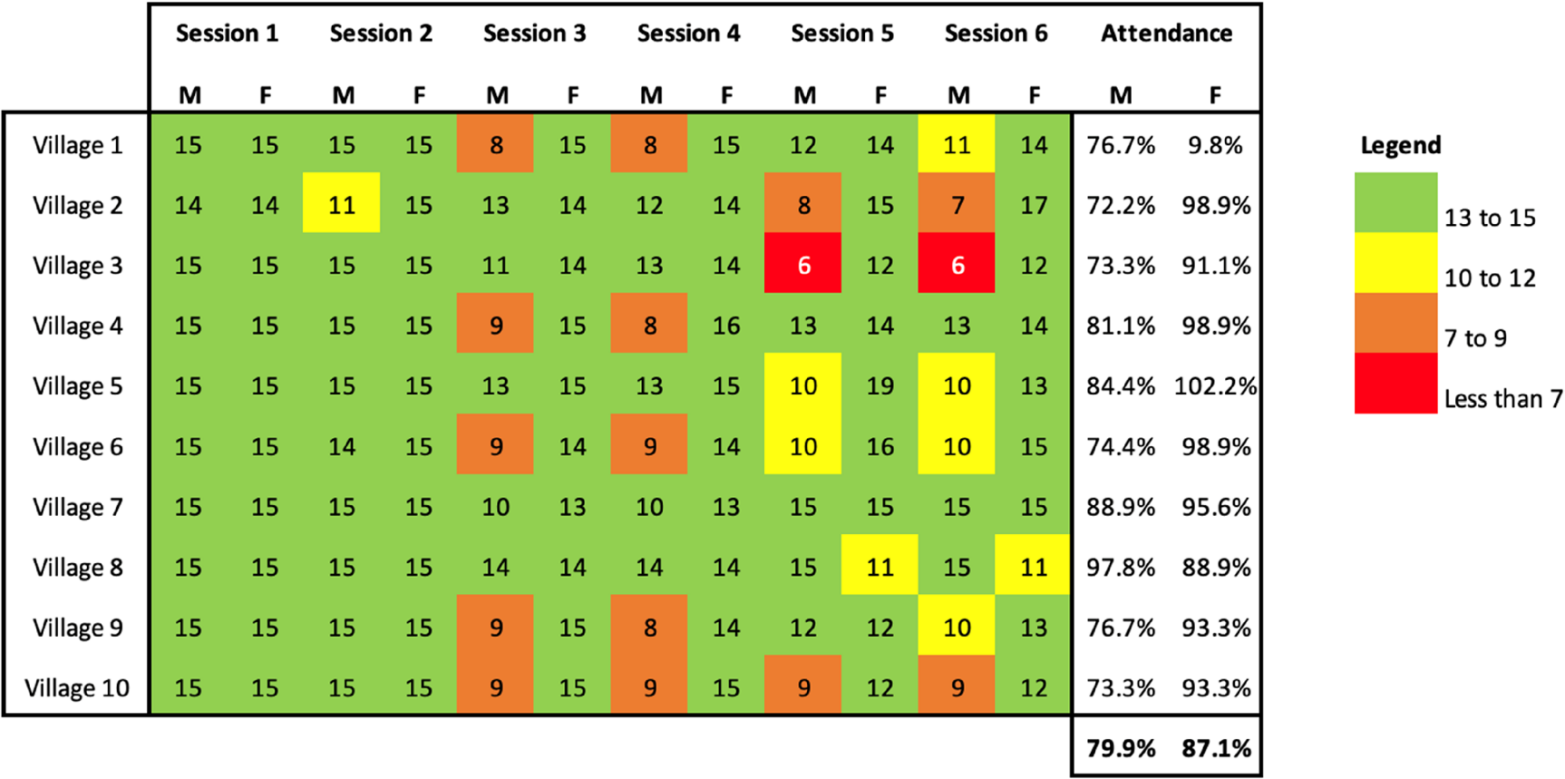
Attendance rates for men and women across the six intervention sessions

As shown in Table 3, there was a decline in male attendance across five of the ten villages for sessions 2 and 3, while male attendance increases again in these villages for sessions 5 and 6. The facilitators explained this as the result of a negative reaction by men in some of the villages to the messages introduced in the first two sessions about gender inequality. For instance, some male participants from village 4 felt that the focus of the session on gender inequality was blaming them for violence and stopped attending. The facilitators and CBRs made a conscious attempt to convince the men that they should return to the sessions, as shown in the uptick in male participation in some villages. However, men’s attendance was patchy by session 6 for most villages for various reasons, including travel overseas and plantation work.

In informal interviews with community members conducted by SVSG staff, participants reported favourable perceptions of the intervention. They mentioned the value of learning about violence and how they had applied the lessons learned in their own lives. For example:

*Aoga tele le polokalame I totonu o si o matou aiga aemaise lava I le ma ulugalii ma lou toalua. O au o se tagata faamaualuga I le ma va. Ae ua aoga le polokalame ua mafai a.i. ona ou iloa le mea e tatau ona fai I totonu o si o ma aiga ina i.a. mafai ona ma nonofo fealofani a.i. ma leo toe tutupu a.i. ni sauaga masani ona tutupu.*

The intervention is very useful in our family, especially for my husband and I. I am a proud person in our family. But the programme has been useful and has allowed me to know what needs to be done in our family so that we can live together in peace and prevent the recurrence of violence that often occurs. (Village 01, woman, 35 years old)

Community participants mentioned the intervention’s focus on including everyone in the village, particularly youth, as a key strength. The social structure of Samoan villages that prioritise separate discussions for youth groups makes this type of intergenerational discussion rare. They saw the intervention as providing a unique opportunity to discuss issues affecting the village intergenerationally. As one participant expressed:

*Ua tele le aoga*,* muamua ua mafai ona tuufaatasia a.i. vaega uma ole nuu e soalaupule faafitauli ma ni auala e fofo a.i.*,* ae e le masani a.i. ona tupu se mea faapea ae faafetai ile polokalame mo le avanoa e feutagai a.i. matai ma tina ma fanau a le nuu.*

It has been very beneficial, firstly it has been able to bring together all ages of the village to discuss problems and find ways to solve them, but this does not happen often, but thanks to the programme for the opportunity to consult with the Matai and the mothers and children of the village. (Village 08, woman, 52 years old)

Many participants highlighted the adaptation of the intervention to the Samoan context and Christian beliefs as a strength. This adaptation was strengthened by the specific backgrounds of the facilitators involved in the intervention (one was the adult daughter of a Church Minister, while the other held a high Chief title in his village). As recognised individuals in Samoan society with complementary strengths, these facilitators were able to expertly navigate two pillars of Samoan society: *fa’a Samoa* (the Samoan way) and Christian beliefs, while maintaining the integrity of the intervention.

An example of a specific adaptation is in the word “intervention,” which has no equivalent term in Samoan. The facilitators’ spent many hours trying to find a way of explaining the transformative potential of interventions versus programmes (which are often perceived as a mechanism for delivering information). They settled on the term *uluulumatafolau*, which has many meanings in the Samoan oratory language. It refers to repairing the thatched roof of a home if it is leaking, which participants felt was apt to describe interventions because you intervene when something is not right. This meaning resonated with participants because of the work of the missionaries in early 1800s who entered homes to spread the Gospel. *Uluulumatafolau* is also used to refer to the process by which the Holy Spirit enters and resides in you, a meaning with highly positive connotations in Samoa. As mentioned by a facilitator, participants had expressed a deep appreciation for the use of this word during sessions:

…both men and women discussed how the interventions had re-shaped their thoughts and behaviours… Translating “intervention” into a spiritual and Samoan oratory word added weight…The word we used for “intervention”—*uluulumatafolau—*has such a deep meaning in the Samoan cultural context that participants felt a deep connection to it. (Facilitator, written discussion via email, 8 Sept 2025)

Moreover, participants did not like the fact that husbands/wives/partners could not be involved in the intervention, demonstrating that participants were engaged in the intervention and saw value from it. The intervention team had decided to allow only one member from each family to participate, to minimise perceptions of unfairness among families in the village. Participants argued that both men and women from the same families needed to be involved so that both members of a couple could make the same changes in the household.

### Feasibility

The intervention was fully implemented according to schedule in all 10 participating villages. CBRs selected members of their community to participate, however, other family members often arrived on the day to replace the selected individual. As shown in Table 4, 26 men (17.3%) and 31 women (20.7%) were replaced with another member of the same family over the course of the intervention. Moreover, discussions with the facilitators in project team meetings highlights the perceived importance of continuity of participants:

I would think that we really need to re-visit our participants and their commitment to the programme. This would have improved our success rate as we would have a continuity of participants from the beginning, with their buy-in to the programme until the end. This, to me, was perhaps the biggest drawback of the programme, having different participants throughout the programme and I know that the intention was there to have the same ones for the duration of the programme, but it did not happen. (Facilitator, written discussion via email, 8 Sept 2025)

Table 4 shows the percentage of participants for 2, 4, and 6 (all) session by village. As inferred by the facilitator in the quote above, attendance differed by village depending on the commitment of CBRs to the intervention in each village. While some CBRs emphasised the need to attend all six sessions, others were more relaxed in the approach they took to ensuring consistency in attendance.

**Table 4:**
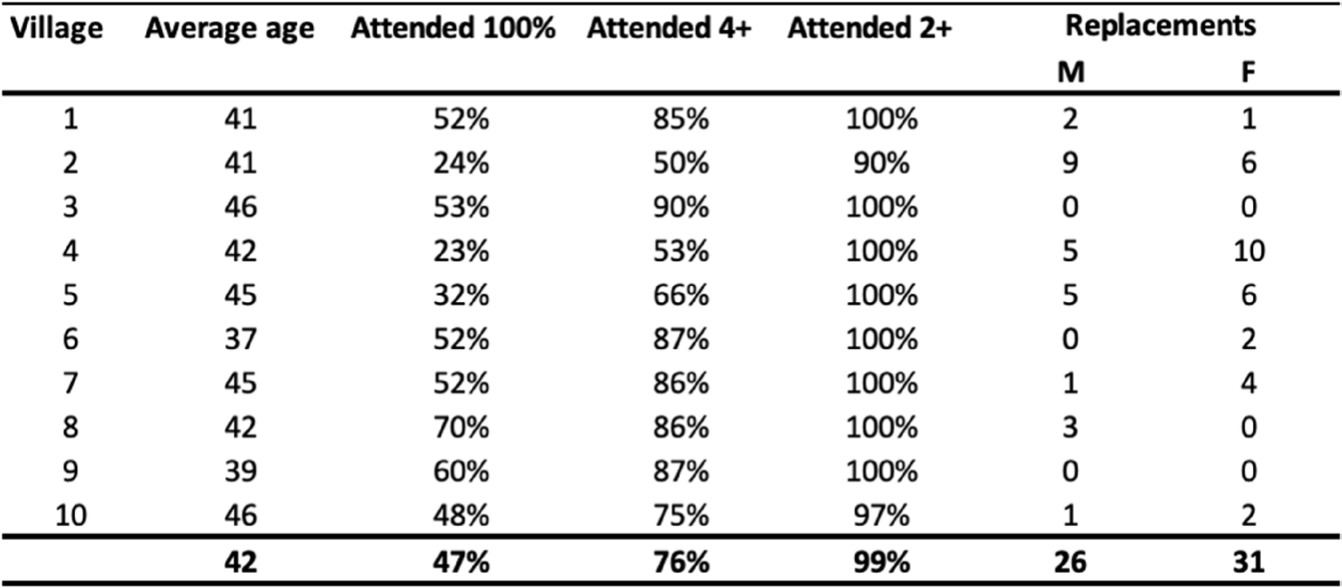
Participant retention and replacements by village

For example, villages 2 and 4 saw both a high number of replacements relative to other villages (15 in both villages), and a reduction in the number who participated in all 6 sessions (24% and 23% respectively). Whether CBRs were committed or not to ensuring regular attendance was not reflected in data on their commitment to the intervention overall. The attendance patterns of CBRs (as an indicator of their commitment to delivering the intervention) is shown in Table 5. The CBRs from villages 2 and 4 were all “very active” or “active” individuals (as assessed by SVSG staff) with a nearly perfect attendance record.

**Table 5:**
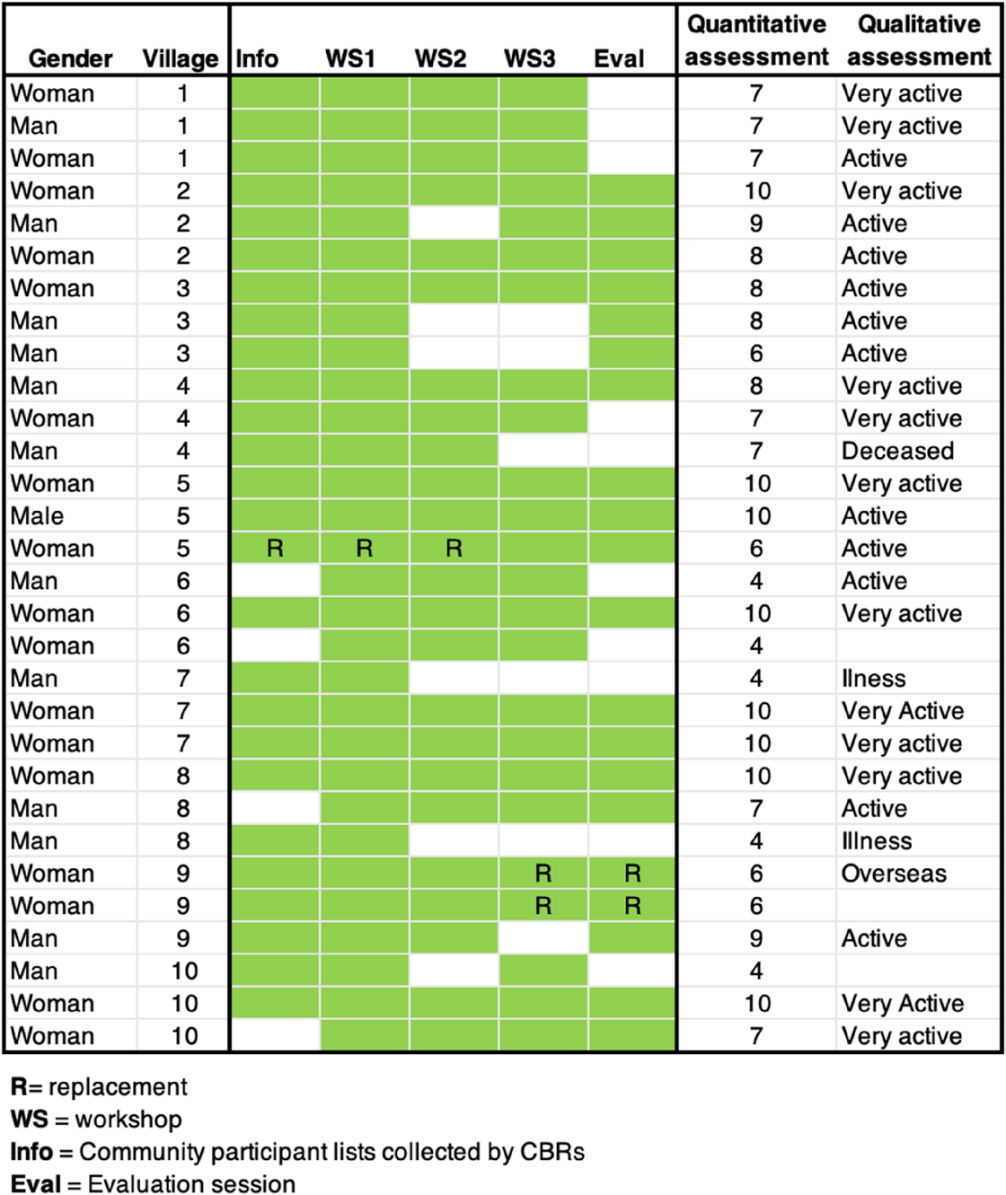
CBR attendance during workshops

The reasons for poor attendance in certain villages likely rests with a combination of factors that we do not have the data to fully explore or control as part of the pilot, i.e., existing relationships and trust between CBRs and community participants, the clarity of the message about the need for full attendance, etc.

The active involvement of CBRs in the intervention co-design brought other advantages. CBR leadership in sample selection meant that CBRs were well placed to motivate community members to attend the intervention and engage local stakeholders. In interviews, CBRs mentioned the commitment of local leaders as a key enabler of the intervention, which had emerged as an implementation strategy in discussion with CBRs in the first place. When asked who attended the intervention from their village, CBRs explicitly mentioned inviting those holding Matai titles, government positions, well-respected elders in their villages, as the individual holding power to bring about change for the village.

*Oute iloa matua fiafia pule mamalu a alii ma faipule aua le poloketi lenei. Ae ole molimau i.a. lena ole matua lagolagoina e afioaga o alii ma faipule.*

Local leaders and government representatives (in our village) are very happy with this project… (The success of the project) is testimony to the strong support of the village council and government representatives. (CBR, male, village 08, Savai’i, 8 Feb 2024)

While the involvement of local leaders demonstrated their commitment to the intervention, strengthening its legitimacy, the project team also observed that this shaped group dynamics during sessions: women, youth and untitled men were far less likely to speak in sessions where Matai were present.

### Potential impact

The surveys indicated a reduction in reported experiences of IPV at endline compared to baseline; however, changes were not statistically significant. We observed a modest yet significant reduction in belief in non-equitable norms among women (β = − 0.54, 95% CI − 0.88 to − 0.21, p = 0.005; Table 6). Among men, reductions in violence perpetration of physical and sexual violence were large and in the correct direction, but these did not reach statistical significance (Table 7).

**Table 6:** Pre/Post Intervention outcomes measures for women

Outcome measures	Baseline *n*(%) or median (IQR)*	Endline *n*(%) or median (IQR)*	Coefficient* aOR or β* (95%CI)	*P*-value
	*N* = 161	*N* = 140		
Experience of physical IPV past 6 months	25 (18.9%)	15 (12.5%)	0.65 (0.36–1.16)	0.142
Experience of sexual IPV past 6 months	9 (6.6%)	10 (8.3%)	1.20 (0.31–4.63)	0.794
Experience of emotional IPV past 6 months	18 (12.9%)	13 (10.9%)	0.86 (0.52–1.42)	0.548
Experience of economic IPV past 6 months	45 (33.6%)	31 (26.7%)	0.76 (0.52–1.11)	0.153
GEM norms score (higher=more inequitable gender norms)	9 (8–11)	9 (8–10)	-0.54 (-0.88- -0.21)	**0.005**
GEM violence score (higher=more accepting of violence)	3 (0–6)	3 (1–5)	0.02 (-0.96-1.00)	0.961

*Adjusted for age, education, and village cluster following evidence of associations with IPV from a larger dataset collected in the same villages [27]. Village was included in adjustments to account for clustering at the village level

Bolded values are statistically significant at a 95% confidence level

**Table 7:** Pre/Post Intervention outcomes measures for men

Outcome measures	Baseline *n*(%) or median (IQR)*	Endline *n*(%) or median (IQR)*	Coefficient* aOR or β* (95%CI)	*P*-value
	*N* = 128	*N* = 89		
Perpetuated physical IPV past 6 months	8 (8.1%)	1 (1.5%)	0.14 (0.01–1.84)	0.134
Perpetuated sexual IPV past 6 months	10 (10.0%)	3 (4.5%)	0.40 (0.10–1.64)	0.203
Perpetuated emotional IPV past 6 months	18 (18.0%)	11 (16.2%)	0.89 (0.43–1.84)	0.755
Perpetuated economic IPV past 6 months	20 (19.8%)	10 (14.7%)	0.70 (0.38–1.30)	0.970
GEM norms score (higher=more inequitable gender norms)	8 (6–10)	8 (6–10)	0.05 (-1.38-1.48)	0.934
GEM violence score (higher=more accepting of violence)	3 (2–5)	3 (0–5)	-0.28 (-1.22-0.68)	0.541

*Adjusted for age, education and village cluster following evidence of associations with IPV from a larger dataset collected in the same villages. Village was included in adjustments to account for clustering at the village level

### Limitations

This study had several limitations. First, this is a pilot and the relatively small sample size limits the statistical power of our analyses and reduces our ability to draw meaningful conclusions about what was observed. In addition, the absence of a control group constrains our ability to assess changes over time relative to a comparable population. Without a counterfactual, it is difficult to determine whether the changes observed can be attributed to the intervention or to other contextual or temporal factors (e.g. policy change). As such, findings should be interpreted with caution and viewed as exploratory rather than definitive.

Second, 60 participants were lost to follow-up at the endline survey in comparison to the baseline survey (an attrition from 289 participants at baseline versus 229 at endline). Other participants were substituted by family members during recruitment when selected individuals were unavailable. However, because detailed records of these substitutions and how replacement participants were selected were not systematically retained, it is not possible to fully assess the extent to which this may have influenced the composition of the baseline and endline samples. While sample composition appears similar at baseline and endline, this may have affected the internal validity of the sample in ways we are unable to detect and which may limit the potential generalisability of our results. It also prevented adjustment for within-person clustering and reduced the precision with which individual-level changes, such as violence experience and perpetration, could be measured.

This points to the need for a larger pilot study of the *E le Saua le Alofa* intervention that maintains detailed records of the characteristics of any replacements to more accurately estimate changes in violence experience/ perpetration at different time points. However, such decisions also need to consider local understandings of recruitment and how it is perceived in light of concepts of family responsibility and obligation. Moreover, employing a one-to-one approach for the survey questionnaire, with field workers asking questions or assisting participants with survey completion, would improve completion rates while leveraging the importance of interpersonal interactions in this and other communitarian contexts.

Third, we did not stratify participants but left the decision about who should participate to the CBRs, who had three years of research training workshops as part of the intervention co-design process. While this training helped to ensure that community participants were distributed across gender, age and village status, achieving perfect balance was not always possible, and women were more prominently represented in the final sample.

## Discussion

The results of the pilot provide preliminary indications of the acceptability and feasibility of the *E le Saua le Alofa* intervention in Samoan communities and suggest possible benefits for participants. These results highlight the potential for carefully co-designed interventions and support the case for further piloting with a larger population. One of the key innovations of this intervention was the long-term (over three years) and intentional adaptation process, which actively incorporated Indigenous knowledge systems and entered into dialogue with *fa’a Samoa* and religious thinking on violence in this context [10]. This is aligned with what Okamoto has referred to as a “deep structure adaptation” in targeting the root causes of IPV, including colonial legacies, capitalist political economy, and gendered power relations [28].

Another key factor contributing to the acceptability and feasibility of the intervention was the involvement of community representatives as CBRs. While the acceptance of the CBR model by community members has been discussed at length elsewhere [29], the results of the pilot demonstrate the importance of the coordination role they provided in the long-term feasibility of the intervention. CBRs were able to access members of their community in a way that the project team and implementing partner (SVSG) could not, encouraging community members to attend intervention activities and emphasising the overall importance of the intervention’s aims for the village. In some villages, CBRs played a key role in encouraging participants to return to the intervention when they thought it not relevant to their lives.

The pilot also highlights modifications that might improve its acceptability and relevance to local Samoan communities. Participants emphasised the importance of working with couples as part of violence prevention interventions, while also acknowledging that couples often live in and are influenced by extended family groups. This would align the intervention more with other evidence-based interventions in the Asia-Pacific region such as *Zindagii Shoista*, which takes an intergenerational approach to addressing violence through involving entire households and emphasising intergenerational conflict and power as a key part of the intervention [20]. A multi-generational, family-oriented approach would align with Pacific understandings of relationality and collective responsibility [30], fostering protective environments that extend beyond the nuclear household and at-risk couples.

Undermining the potential acceptability of the intervention, nearly half of the villages saw reductions in men’s participation following a session on addressing power relationships. This points to the need for careful calibration of gender equality sessions to local understandings of gender roles in relationships. While addressing gender norms is central to the intervention’s theory of change (which was developed by CBRs), how gender is framed in intervention delivery must resonate with Samoan cultural values and norms. This requires further work with local stakeholders to understand potential points of leverage within Samoa’s religious institutions, building on the important work of Ah Siu-Maliko and existing frameworks for anti-violence work within the Christian tradition [31].

Additionally, the pilot highlights changes needed not only in the intervention but in *how* we measure it, so that evaluation methods are aligned with Indigenous epistemologies. Standard surveys and randomised processes can feel alienating and extractive – separating evaluators from communities in ways that undermine trust and local knowledge. Although data is limited, we suspect that this may be a reason why some participants disengaged from the endline survey. Several suggestions have been made by Indigenous scholars to improve survey methodologies offer innovative opportunities for participants to situate answers within story and context, including embedding brief survey items inside natural conversational approaches (i.e., talanoa, yarning or talk-story) [32, 33]. Such an approach, which keep the structured portion to a minimum reduces survey fatigue, warrants further investigation.

## Conclusion

Overall, our findings suggest that interventions in Pacific contexts are most promising when they combine evidence-based strategies with culturally grounded adaptation, multi-generational engagement, and locally embedded facilitation and evaluation. Future research should continue to refine these approaches, particularly regarding gender norm discussions and survey methodologies, to ensure both fidelity and cultural resonance of *E le Saua le Alofa*.

## Supplementary Information

Below is the link to the electronic supplementary material.

Supplementary Material 1.

Supplementary Material 2.

## Data Availability

The data underlying this article will be shared on reasonable request to the corresponding author.
