# Supplementary material for: E le Saua le Alofa (Love shouldn’t hurt): exploring the acceptability, feasibility and potential impact of a co-developed intervention to prevent violence against women in Samoa
Source: BMC Public Health. 2026 Mar 24;26:1432. doi: 10.1186/s12889-026-27013-z (PMC13134231; doi:10.1186/s12889-026-27013-z)
Supplement: Supplementary file 1 — Supplementary Material 1. [file 12889_2026_27013_MOESM1_ESM.pdf]

E le Saua le Alofa **(Love Shouldn't Hurt): Evaluating the acceptability, feasibility and potential impact of a co-developed intervention to prevent violence against women in Samoa**

Supplementary file - Topic guide for interviews with community-based researchers

---

Thank you for sitting down with me to discuss this. The questions I want to ask about the project are really about what changed. If nothing changed, that's absolutely fine. It is also really important to hear about what didn't work so we can fix things for the future.

If there are any question I ask that you don't want to answer, just let me know and we can skip these.

1. Community change
   - Were there any changes in your village that came out of the project?
   - Was the village council or any matai involved in the project? What did they do/ say?
   - Who else was involved?
   - Did people change their beliefs or views about violence? Can you give me an example?
   - Do people in your community come to see you when a someone is in need? Did they do this before the project, or has it changed?
   - Have there been any cases of violence in your community since the start of the project? If so, what happened? What did you do in response?

2. Individual change
   - Have you enjoyed being part of the project? What is it that you have enjoyed most?
   - Has being part of the EVE Team helped you personally? Can you give me an example?
   - Has being part of the project had any impact on your own personal situation? In what way?
   - Have your thoughts or ideas about violence changed as a result of participating in the project? Can you give me an example?
   - If someone told you 'this woman deserves the violence,' would you say something? What would you say to them?

3. Logistics/ attendance
   - Did you participate during the village meetings? It is fine if you didn't, we just want to know the reasons why?
   - Why do you think people dropped out of the project over time?
   - If we didn't pay people, do you think they would still have come?
   - There were more women that participated than men throughout the project. Any thoughts on why this might be the case?

Thank you for taking the time to talk to me. We really appreciate the time and effort you have put into the EVE Project over the years, and hope we will be able to continue.

The information you have provided will be really helpful for the evaluation.
