# Supplementary material for: E le Saua le Alofa (Love shouldn’t hurt): exploring the acceptability, feasibility and potential impact of a co-developed intervention to prevent violence against women in Samoa
Source: BMC Public Health. 2026 Mar 24;26:1432. doi: 10.1186/s12889-026-27013-z (PMC13134231; doi:10.1186/s12889-026-27013-z)
Supplement: Supplementary file 2 — Supplementary Material 2. [file 12889_2026_27013_MOESM2_ESM.docx]

***E le Saua le Alofa* (Love Shouldn’t Hurt): Evaluating the acceptability, feasibility and potential impact of a co-developed intervention to prevent violence against women in Samoa**

**Supplementary file**: Intervention components

| **Intervention component** | **Driver of violence (from ToC)** | **Learning objectives** | **Core topics** | **Source intervention** |
| --- | --- | --- | --- | --- |
| **Session 1 - Addressing gendered power relations** | Abuse of power by men | To explore power in relationships and what it means to be controlled. To collaborative define what power and control look like. | - Expectations of men/ women - Giving advice to others | Stepping Stones (South Africa) |
| **Session 1 - Addressing gendered power relations** | Gendered social norms | To explore images and realities of the ideal man and woman and how these are shaped by structural/social factors. | - Explanation of power (power over, power to, power with) | MAISHA (Tanzania) |
| **Session 2 - Achieving healthy violence-free relationships** | Family conflict, arising from poor communication | To provide practical skills for reducing violence in relationships. | - Triggers of violence - Sharing positive time with partners | EMAP (DRC)  Indashyikirwa (Rwanda) |
| **Session 3: Knowing ourselves and communicating effectively** | Poor communication between couples | To discuss the risks and consequences of alcohol abuse with men and how to develop strategies for mutual support.  To empower assertive communication by women. | - Risks and consequences of alcohol abuse (men) - I-statements (women) - Assertive communication (women) | Bandebereho (Rwanda)  MAISHA (Tanzania)  Stepping Stones (South Africa) |
| **Session 4: Positive parenting** | Family conflict, arising from previous experiences of child abuse | To help participants explore different types of parenting, and the impact of discipline and punishment on our children. | - Different kinds of parenting - The difference between discipline and punishment - Positive parenting skills | Parenting for Respectability (Uganda) |
| **Session 5: Supporting livelihoods** | Family conflict, arising from economic stress | To develop skills in coping with crises and identifying personal goals.  To identify viable, accessible business opportunities and the resources needed. | - Resources needed to sustain livelihoods - Income generating activities | Stepping Stones and Creating Futures (South Africa) |
| **Session 6: Supporting survivors** | Gendered social norms | To build understand of how fear and stigma can prevent survivors from seeking help.  To empower participants to be an ally to survivors. | - How fear and abuse can prevent help-seeking - How to help women in abusive relationships | Zindagii Shoista (Tajikistan)  Indashyikirwa (Rwanda) |
